# Attitudes toward lithium prescription among psychiatrists in Saudi Arabia: a cross-sectional study

**DOI:** 10.3389/fpsyt.2025.1609038

**Published:** 2025-06-19

**Authors:** Mohammad A. Alolayan, Ahmad H. Almadani, Ghada K. Alrashed, Jamal Alothaim, Weam Hussein

**Affiliations:** ^1^ Department of Psychiatry, College of Medicine, King Saud University, Riyadh, Saudi Arabia; ^2^ SABIC Psychological Health Research and Applications Chair (SPHRAC), Department of Psychiatry, College of Medicine, King Saud University, Riyadh, Saudi Arabia; ^3^ Department of Psychiatry, King Saud University Medical City, King Saud University, Riyadh, Saudi Arabia; ^4^ Academic Affairs, College of Medicine, King Saud University, Riyadh, Saudi Arabia

**Keywords:** lithium, bipolar disorder, psychiatrists, residents, attitude, prescription, Saudi Arabia

## Abstract

**Background:**

Bipolar disorder (BD) is a mental illness with an estimated overall lifetime prevalence of 2.4% worldwide. Various pharmacological agents are available for treating BD, one of which is lithium. Lithium is commonly recommended as a first-line treatment during the maintenance phase of BD. However, prescribing patterns for mood disorders vary among countries, with notable regional differences in lithium use.

**Methods:**

This cross-sectional study included 287 psychiatrists and psychiatry trainees in Saudi Arabia. A survey consisting of 22 multiple-choice questions was sent via WhatsApp to all participants. Data were collected between January and May, 2024.

**Results:**

Lithium was prescribed to BD patients by 72.5% of the participants. Most participants (70.7%) prescribed lithium to up to 25% of their patients. The primary reasons for not prescribing lithium were concerns about its adverse effects (64.8%), followed by the need for monitoring (53.7%), and the unavailability/shortage of lithium supply in Saudi Arabia (45.6%). Psychiatrists working in general hospitals (p=0.017) were more likely to prescribe lithium to patients with BD. In contrast, psychiatrists with limited experience or those who were unfamiliar with lithium treatment (p=0.001) were less likely to prescribe lithium.

**Conclusion:**

The use of lithium in Saudi Arabia is often influenced by concerns about its side effects, the need for monitoring, and product availability. Psychiatrists’ familiarity with lithium and the settings in which they practice are significant factors shaping prescribing behavior. Future efforts should focus on addressing the barriers to lithium prescription, including enhancing clinician training and improving access to lithium.

## 1 Introduction

Bipolar disorder (BD) is a chronic mood disorder with an estimated overall lifetime prevalence ranging from 0.49% to 1.0% (1, 2), with an additional 1.4% of individuals meeting the criteria for subthreshold bipolar states (2). The Saudi National Mental Health Survey reported that the lifetime prevalence of BD in Saudi Arabia was 3.2% (3). However, this estimate is relatively high and may reflect the use of lay-administered World Health Organization Composite International Diagnostic Interview, which may have led to the inclusion of false-positive cases.

Lithium was discovered by the Australian psychiatrist John Cade (4). In 1970, the United States Food and Drug Administration authorized the use of lithium in the treatment of acute mania, and in 1978, it was authorized for the maintenance treatment of BD (5). Most international guidelines support the use of lithium as a first-line treatment for BD during the maintenance phase (6, 7). Moreover, studies have shown that lithium has anti-suicidal properties in addition to its mood-stabilizing properties (8). This property of lithium is relevant because up to 15% of patients with BD commit suicide (9).

A study assessing clinicians’ attitudes toward lithium use in 43 countries showed that 59% of the respondents chose lithium as the most preferred treatment option for the maintenance treatment in BD (10). However, a study published in 2020 found a decrease in the prescription of lithium and other mood stabilizers for the treatment of patients with BD. The reduction in lithium prescription has been accompanied by a substantial increase in the use of atypical antipsychotics over the last two decades (11). A Swedish study reported that the prescription rate of lithium in BD decreased from 51% in 2007 to 41% in 2013 (12). The prescription rate of lithium for BD in Germany was found to be as low as 26.2% (13). In a Canadian cross-sectional study, 37.9% of participants preferred second-generation antipsychotics for BD maintenance compared to 31% who preferred lithium (14). A study assessing international prescribing patterns for mood disorders revealed significant regional differences in lithium prescription (15), and another revealed variance between countries (16).

Compared with other mood stabilizers, lithium requires more regular tests and assessments (e.g., blood plasma levels, renal profiles, thyroid function tests, and electrocardiograms) (17). Lithium prescriptions have decreased, possibly because of its narrow therapeutic index, cumbersome monitoring requirements, and adverse effect profile (18, 19). However, it is unclear whether these factors have led to the recent steady decline in lithium prescription in various countries (11, 12, 20).

To the best of our knowledge, no previous studies have considered clinicians’ attitudes toward lithium prescription in Saudi Arabia. Therefore, in this study, we aimed to identify the patterns of lithium prescription across Saudi Arabia and highlight the main reasons for under-prescription of lithium in the country.

## 2 Methodology

### 2.1 Study population, recruitment, and sampling

All board-certified psychiatrists and psychiatry residents practicing in Saudi Arabia were the targeted population for this study. An estimated 1,350 practitioners are registered with the Saudi Commission for Health Specialties (21). The estimated sample size was 300 based on a 5% margin of error and 95% confidence interval (CI). The survey was disseminated through WhatsApp groups of psychiatrists and trainees in all regions of the country, who were encouraged to share it with their colleagues. Data were collected through an anonymous questionnaire sent via WhatsApp to the target population using convenience sampling from January to May, 2024.

### 2.2 Survey instrument

The survey was adapted from a study conducted in 2021 (22). The survey was open-access, and the authors’ permission to use their questionnaires was obtained. The survey was a self-administered questionnaire consisting of 22 questions; the first 7 questions covered participants’ demographics, and the last 15 concerned key aspects of lithium use, including indication, prescription rate, dosage frequency, monitoring, and treatment options for BD. The original questionnaire was slightly modified and some questions were replaced to suit the unique characteristics of the study population. The changes included the regions where the participants worked, years of practice, participants’ classification in the Saudi Commission for Health Specialties (SCFHS, e.g., consultant or resident), and the sector in which the participants predominantly worked. The response “unavailability/shortage of supply of lithium in Saudi Arabia” was added regarding the main reasons for not prescribing lithium. The survey was conducted using SurveySparrow (https://surveysparrow.com).

### 2.3 Ethics

Ethical approval for this study was obtained from the King Saud University Institutional Review Board (E-23-8228). Participation was voluntary, and the introductory page of the survey provided informed consent. No identifying information was collected, and the collected data were kept confidential and accessible only to the researchers.

### 2.4 Statistical analysis

Categorical variables are described as counts and proportions (%). The relationship between positive attitudes toward lithium prescription in patients with BD according to sociodemographic characteristics and the perceived reason for not prescribing lithium was determined using the chi-square test. Significant results were tested using a multivariate regression model to determine significant independent factors of positive attitudes toward lithium prescription, with corresponding odds ratios (OR) and 95% CIs. Statistical significance was set at p<0.05. All statistical data were analyzed using Statistical Packages for Social Sciences version 26 (Armonk, NY: IBM Corp., USA).

## 3 Results

This study enrolled 287 psychiatrists and psychiatry trainees. As presented in **Table 1**, 43.6% of participants were aged 25–35 years, and the majority were male (61.7%). Most participants had finished their psychiatry residency training (75.3%). Psychiatrists in the Central Region constituted 39% of participants, and 30.7% had 6–15 years of practice experience. Most participants worked in the public sector (88.9%), and 39% worked in general hospitals.

**Table 1:** Socio-demographic characteristics of the psychiatrists ^(n=287)^.

Study variables	N (%)
Age group	
· 25–35 years	125 (43.6%)
· 36–45 years	79 (27.5%)
· 46–55 years	42 (14.6%)
· 56–65 years	32 (11.1%)
· >65 years	09 (03.1%)
Gender	
· Male	177 (61.7%)
· Female	110 (38.3%)
Classification in Saudi Commission for Health Specialties (SCFHS)	
· Psychiatry Consultant	125 (43.6%)
· Psychiatry Senior Registrar	65 (22.6%)
· Psychiatry Registrar	26 (09.1%)
· Psychiatry Resident	71 (24.7%)
Region of practice	
· Central Region	112 (39.0%)
· Western Region	77 (26.8%)
· Eastern Region	54 (18.8%)
· Southern Region	29 (10.1%)
· Northern Region	15 (05.2%)
Years practicing as a psychiatrist since finishing residency training	
· ≤5 years	65 (22.6%)
· 6–15 years	88 (30.7%)
· 16–35 years	48 (16.7%)
· >35 years	29 (10.1%)
· Still in a psychiatry residency training program	57 (19.9%)
Please indicate in which sector you predominantly provide your services	
· Public	255 (88.9%)
· Private	32 (11.1%)
Please indicate what type of health center you work at	
· Outpatient Clinics	72 (25.1%)
· General Hospital	112 (39.0%)
· Psychiatric Institutions	99 (34.5%)
· All of the above	04 (01.4%)

**Figure 1:**
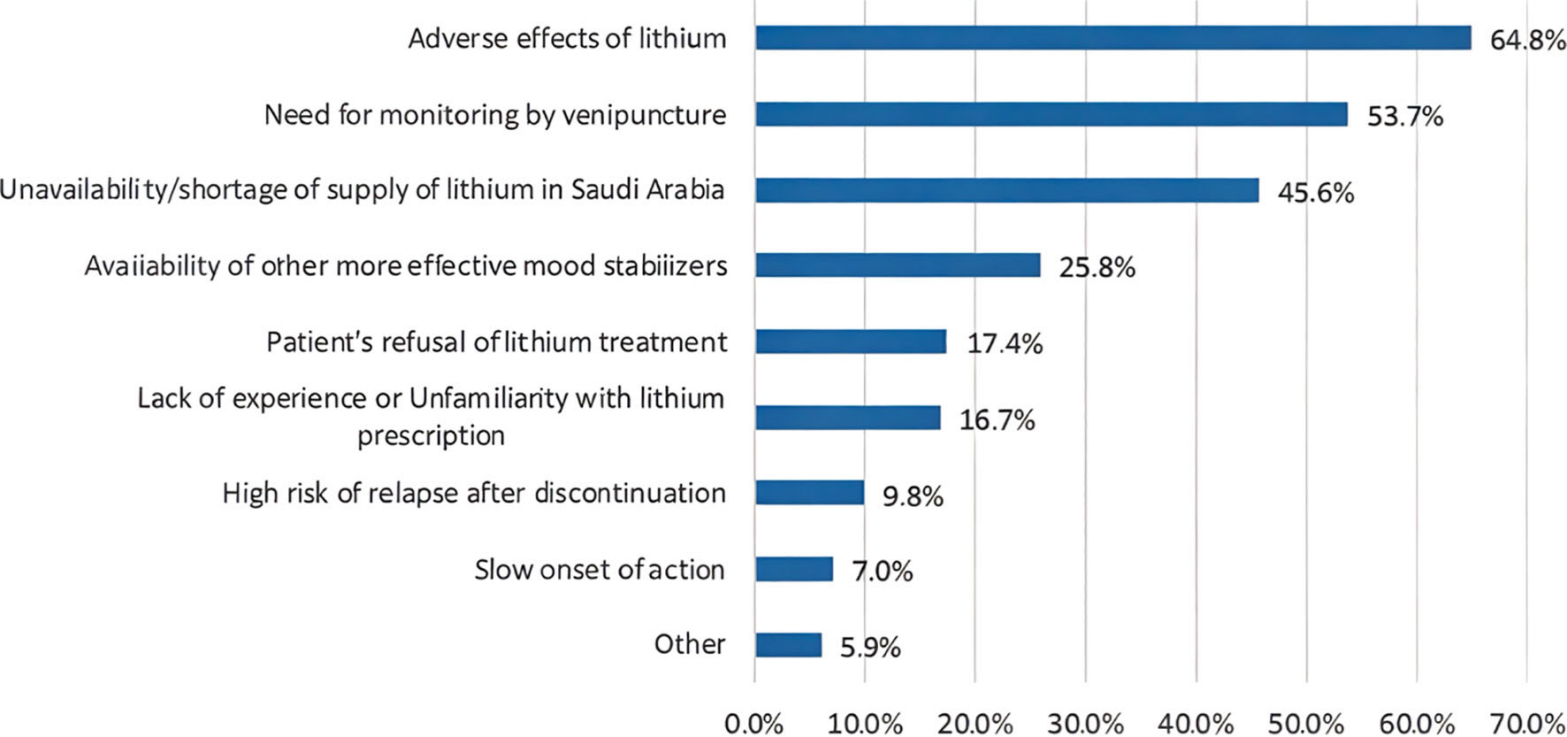
Perceived reason not to prescribe lithium in BD patients.

**Figure 2:**
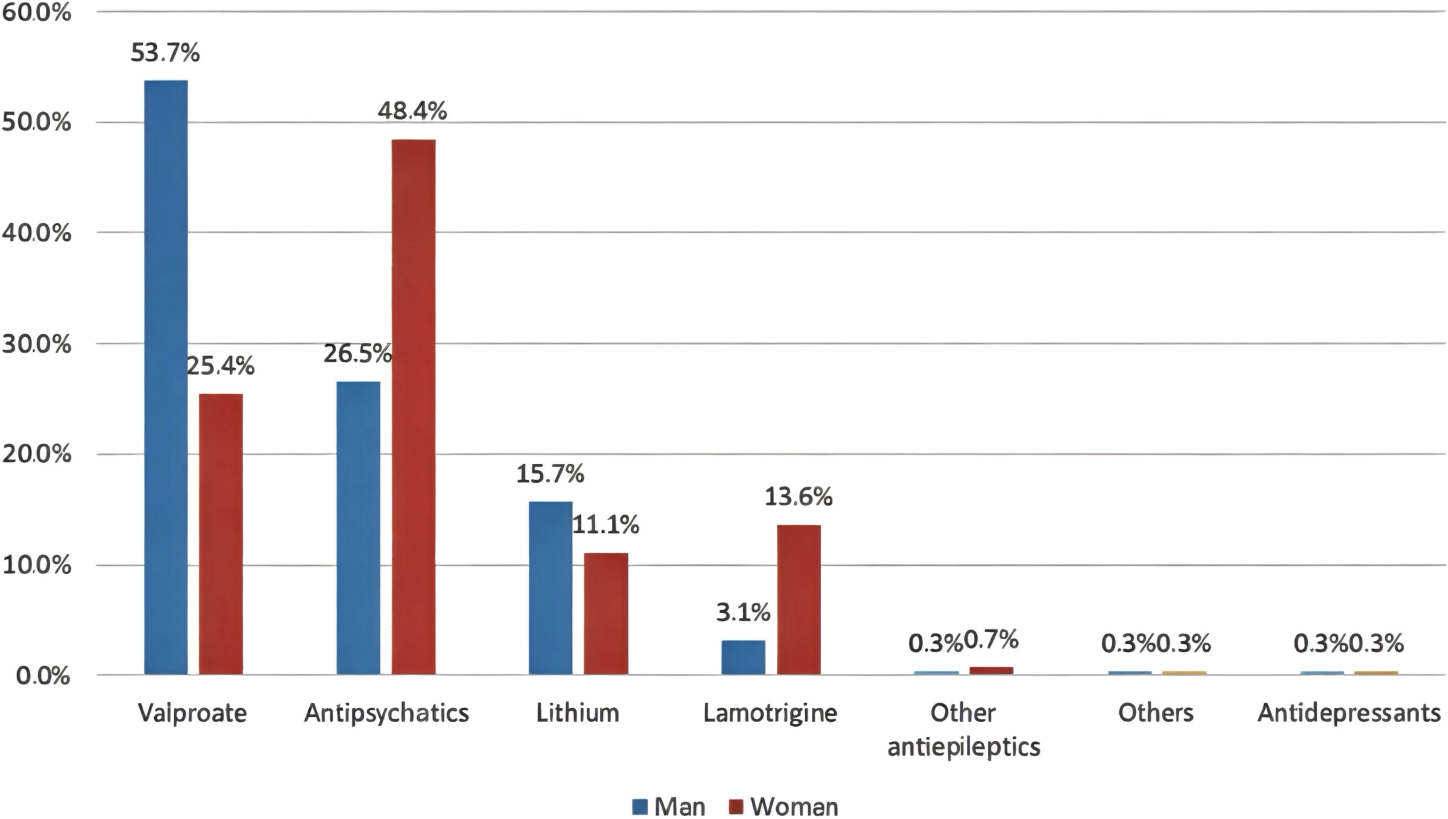
First options for the maintenance treatment of BD in men and women.

**Figure 3:**
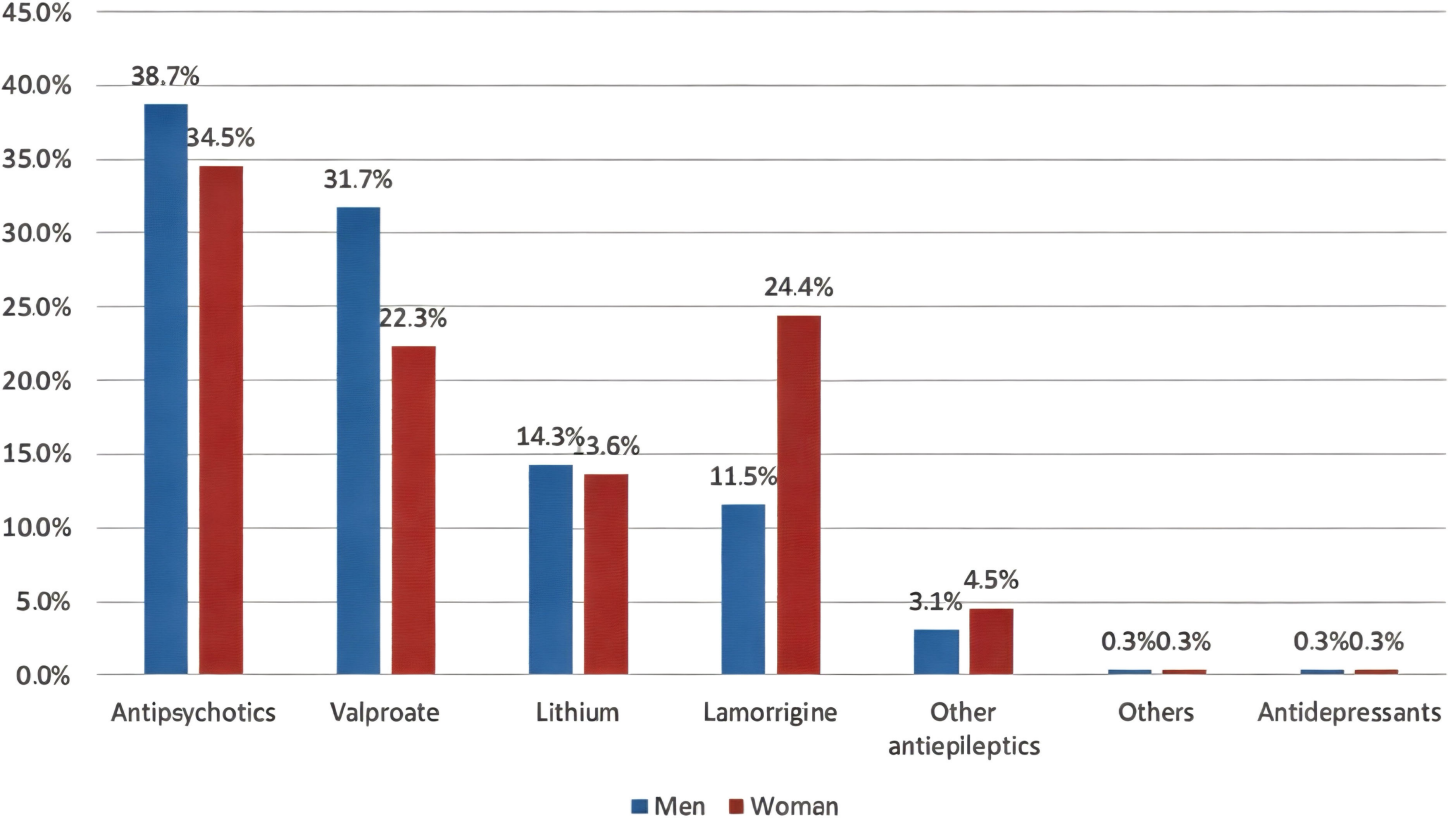
Second options for the maintenance treatment of BD in men and women.

**Table 2** shows participants’ attitudes toward lithium prescription. As many as 72.5% participants prescribed lithium for patients with BD. Of these, 70.7% prescribed lithium to between 0% and 25% of patients. Approximately 31% prescribed lithium after the first manic episode. The “other” option was chosen by 7.3% of participants for the question, “At what point in the course of the illness do you usually prescribe lithium for the maintenance treatment of BD?” Most of them responded with, “when other mood stabilizer fails.” Other answers included “depending on number or relapses,” “according to presenting symptoms,” “difficult cases with suicide risk,” “family history of lithium response,” and “patient profile and preference.” The most common range of serum lithium used for the BD maintenance phase was 0.6–0.8 mmol/L (39.7%). Lithium prescription dosages were usually twice per day (54.7%). Prescriptions to minors, older adults, those with comorbid substance use disorders, and those with comorbid personality disorders were administered by 11.8%, 32.8%, 26.8%, and 59.6% of participants, respectively. The majority of participants followed protocol for monitoring lithium treatment and its adverse effects (69.7%). The most common reasons for not prescribing lithium to BD patients were concerns about its adverse effects (64.8%), the need for monitoring by venipuncture (53.7%), and the unavailability/shortage of lithium in Saudi Arabia (45.6%) (**Figure 1**). As many as 17 participants (5.9%) chose “other,” and their reasons for not prescribing lithium included unavailability of lithium serum level, poor adherence to follow-ups and laboratory tests, patients’ demographics (e.g., women of childbearing age and children), contraindications due to drug interactions, and poor compliance. Furthermore, antipsychotics (48.4%) were the most common first option for the treatment of women with BD, while valproate (53.7%) was the most common first option for men with BD (**Figure 2**). Antipsychotics (women, 34.5%; men 38.7%) were the most common second treatment option for BD maintenance in both sexes (**Figure 3**).

**Table 2:** Assessment of attitudes toward lithium prescription ^(n=287)^.

Attitude item	N (%)
Do you prescribe lithium for patients with bipolar disorder (BD)?	
· Yes	208 (72.5%)
· No	79 (27.5%)
If so, what percentage of patients with BD do you prescribe lithium? ^(n=208)^	
· 0%–25%	147 (70.7%)
· 26%–50%	49 (23.6%)
· 51%–75%	08 (3.8%)
· 76%–100%	04 (1.9%)
At what point in the course of the illness do you usually prescribe lithium for the maintenance treatment of BD?	
· After the first manic episode	89 (31.0%)
· After the first depressive episode, when there is a family history of BD	15 (5.2%)
· In the two previous situations	37 (12.9%)
· During the first 5 years of the illness	42 (14.6%)
· During the first 5–10 years of the illness	11 (3.8%)
· I do not prescribe lithium	72 (25.1%)
· Others	21 (7.3%)
What range of serum lithium levels do you use for the maintenance phase of BD?	
· 0.4–0.6 mmol/L	27 (9.4%)
· 0.6–0.8 mmol/L	114 (39.7%)
· 0.8–1 mmol/L	35 (12.2%)
· 1–1.2 mmol/L	08 (2.8%)
· Anywhere within the 0.6–1.2 mmol/L range	54 (18.8%)
· I do not prescribe lithium	48 (16.7%)
· Other	01 (0.30%)
What is your usual dosing of lithium prescriptions?	
· Once a day in the morning	22 (7.7%)
· Once a day in the evening	68 (23.7%)
· Twice a day	157 (54.7%)
· Three times a day	17 (5.9%)
· Other	04 (1.4%)
· I do not prescribe lithium	19 (6.6%)
Do you prescribe lithium to minors with bipolar disorder (BD)?	
· Yes	34 (11.8%)
· No	130 (45.3%)
· I do not treat minors	123 (42.9%)
Do you prescribe lithium to older adults with bipolar disorder (BD)?	
· Yes	94 (32.8%)
· No	134 (46.7%)
· I do not treat older adults	59 (20.6%)
Do you prescribe lithium to patients with BD and a comorbid substance use disorder?	
· Yes	77 (26.8%)
· No	124 (43.2%)
· I do not treat patients with a substance use disorder	86 (30.0%)
Do you prescribe lithium to patients with BD and a comorbid personality disorder?	
· Yes	171 (59.6%)
· No	93 (32.4%)
· I do not treat patients with a personality disorder	23 (8.0%)
Do you follow official protocol for monitoring lithium treatment and its adverse effects	
· Yes	200 (69.7%)
· No	36 (12.5%)
· I do not prescribe lithium	51 (17.8%)
What is the main reason not to prescribe lithium in BD? *	
· Adverse effects of lithium	186 (64.8%)
· Need for monitoring by venipuncture	154 (53.7%)
· Unavailability/shortage of supply of lithium in Saudi Arabia	131 (45.6%)
· Availability of other more effective mood stabilizers	74 (25.8%)
· Patient’s refusal of lithium treatment	50 (17.4%)
· Lack of experience or unfamiliarity with lithium prescription	48 (16.7%)
· High risk of relapse after discontinuation	28 (9.8%)
· Slow onset of action	20 (7.0%)
· Other	17 (5.9%)
What treatment do you use as the first option for the maintenance treatment of bipolar disorder (BD) in women?	
· Antipsychotics	139 (48.4%)
· Valproate	73 (25.4%)
· Lamotrigine	39 (13.6%)
· Lithium	32 (11.1%)
· Other antiepileptics	02 (0.70%)
· Antidepressants	01 (0.30%)
· Other	01 (0.30%)
What treatment do you use as the second option for the maintenance treatment of bipolar disorder (BD) in women?	
· Antipsychotics	99 (34.5%)
· Lamotrigine	70 (24.4%)
· Valproate	64 (22.3%)
· Lithium	39 (13.6%)
· Other antiepileptics	13 (4.5%)
· Antidepressants	01 (0.30%)
· Other	01 (0.3%)
What treatment do you use as the first option for the maintenance treatment of bipolar disorder (BD) in men?	
· Valproate	154 (53.7%)
· Antipsychotics	76 (26.5%)
· Lithium	45 (15.7%)
· Lamotrigine	09 (3.1%)
· Antidepressants	01 (0.30%)
· Other antiepileptics	01 (0.30%)
· Other	01 (0.30%)
What treatment do you use as the second option for the maintenance treatment of bipolar disorder (BD) in men?	
· Antipsychotics	111 (38.7%)
· Valproate	91 (31.7%)
· Lithium	41 (14.3%)
· Lamotrigine	33 (11.5%)
· Other antiepileptics	09 (3.1%)
· Antidepressants	01 (0.30%)
· Other	01 (0.30%)

* Variable with multiple response answers.

Assessment of the relationship between lithium prescription according to sociodemographic characteristics and perceived reasons for not prescribing lithium to patients with BD showed that participants practicing in general hospitals (p=0.017) and those with patients who refused lithium treatment (p=0.001) were more likely to prescribe lithium. However, participants lacking experience or those unfamiliar with lithium treatment (p=0.001) were less likely to prescribe lithium to patients with BD. No significant relationships were observed between lithium prescription and age, sex, SCFHS classification, region of practice, years of practice, or sector of work (p>0.05) (**Table 3**).

**Table 3:** Relationship between prescription of lithium for patients with BD, socio-demographic characteristics, and perceived reasons for not prescribing lithium to BD patients ^(n=287)^.

Factor	Prescription of Lithium		P-value §
	Yes N (%) ^(n=208)^	No N (%) ^(n=79)^	
Age group			
· ≤35 years	97 (46.6%)	28 (35.4%)	0.088
· >35 years	111 (53.4%)	51 (64.6%)	
Gender			
· Male	130 (62.5%)	47 (59.5%)	0.640
· Female	78 (37.5%)	32 (40.5%)	
Classification in Saudi Commission for Health Specialties (SCFHS)			
· Psychiatry consultants	89 (42.8%)	36 (45.6%)	0.671
· Psychiatry senior registrar, registrar, and residents	119 (57.2%)	43 (54.4%)	
Region of practice			
· Inside Central Region	129 (62.0%)	46 (58.2%)	0.556
· Outside Central Region	79 (38.0%)	33 (41.8%)	
Years practicing as a psychiatrist since finishing residency training *			
· ≤5 years	41 (25.8%)	24 (33.8%)	0.459
· 6–15 years	63 (39.6%)	25 (35.2%)	
· >15 years	55 (34.6%)	22 (31.0%)	
Please indicate in which sector you predominantly provide your services			
· Public	185 (88.9%)	70 (88.6%)	0.936
· Private	23 (11.1%)	09 (11.4%)	
Please indicate what type of health center you work at			
· Outpatient Clinics	42 (20.2%)	30 (38.0%)	0.017 **
· General Hospital	84 (40.4%)	28 (35.4%)	
· Psychiatric Institutions	79 (38.0%)	20 (25.3%)	
· Other	03 (01.4%)	01 (01.3%)	
Perceived reason not to prescribe lithium to BD patients ^†^			
· Availability of more effective mood stabilizers	48 (23.1%)	26 (32.9%)	0.089
· Adverse effects of lithium	139 (66.8%)	47 (59.5%)	0.245
· Slow onset of action	16 (07.7%)	04 (05.1%)	0.435
· Need for monitoring by venipuncture	117 (56.3%)	37 (46.8%)	0.153
· Patient’s refusal of lithium treatment	46 (22.1%)	04 (05.1%)	0.001 **
· High risk of relapse after discontinuation	24 (11.5%)	04 (05.1%)	0.099
· Unavailability/shortage of lithium supply in Saudi Arabia	92 (44.2%)	39 (49.4%)	0.435
· Lack of experience or unfamiliarity with lithium prescription	25 (12.0%)	23 (29.1%)	0.001 **

*Excluded participants undertaking a psychiatry residency training program.

^†^Variable with multiple response answers.

^§^P-value has been calculated using the Chi-square test.

**Significant at p<0.05 level.

Based on the significant results generated in the cross-tabulation, a multivariate regression analysis was subsequently performed (**Table 4**) to determine the significant independent predictors of lithium prescription. The results revealed that, compared to those working in outpatient clinics, participants working in general hospitals were predicted to be at least 2.8 times more likely to prescribe lithium to patients with BD (adjusted odds ratio [AOR]=2.782; 95% CI=1.369–5.652; p=0.005). As expected, participants whose BD patients refused lithium treatment were 4.8 times more likely to prescribe lithium (AOR=4.812; 95% CI=1.642–14.100; p=0.004). Furthermore, participants lacking experience or familiarity with lithium prescriptions were less likely to prescribe lithium to patients with BD (AOR=0.379; 95% CI=0.189–0.760; p=0.006).

**Table 4:** Multivariate regression analysis to determine the significant independent factor of lithium prescription in BD patients ^(n=287)^.

Factor	AOR	95% CI	P-value
Please indicate what type of health center you work at			
· Outpatient Clinics	Ref		
· General Hospital	2.782	1.369 – 5.652	0.005 **
· Psychiatric Institutions	1.518	0.766 – 3.009	0.231
Patient’s refusal of lithium treatment			
· No	Ref		
· Yes	4.812	1.642 – 14.100	0.004 **
Lack of experience or unfamiliarity with lithium prescription			
· No	Ref		
· Yes	0.379	0.189 – 0.760	0.006 **

AOR, Adjusted Odds Ratio; CI, Confidence Interval.

**Significant at p<0.05 level.

## 4 Discussion

In this study, we evaluated the attitudes and practices of psychiatrists regarding lithium prescription for patients with BD in Saudi Arabia. The results revealed a relatively high rate of lithium prescription, with 72.5% of participants indicating that they prescribe lithium to patients with BD. However, the frequency of prescription varied significantly, as most (70.7%) only prescribed lithium to 0–25% of patients with BD. These percentages reflect the low prescription rate of lithium for patients with BD in Saudi Arabia. The shown percentage is lower than findings from Spain, where 70% of survey respondents prescribed lithium to more than 50% of patients with BD (22). The lithium prescription rate for patients with BD in Saudi Arabia is also lower than that in other countries. In the Netherlands, 70% of patients with BD or schizoaffective disorders were treated with lithium (23), whereas in Sweden and Denmark, the prescription rates for BD were 55% and 41.7%, respectively (12, 24). However, the lithium prescription rate in Saudi Arabia is closer to the lower rates observed in Germany (26.2%) and Scotland (22%) (13, 25).

A major reason for not prescribing lithium identified in our study was concern about its adverse effects, reported by 64.8% of participants. This finding is consistent with a Spanish study in which 62% of psychiatrists reported that the primary reason for not prescribing lithium was its side effects (22). This finding mirrors those of other studies in which adverse effects such as weight gain, renal complications, and thyroid dysfunction were identified as deterrents in lithium prescriptions (26–28). In contrast, a study that included 42 countries found that the most common reason for not choosing lithium as the preferred option was patients’ negative beliefs or attitudes toward the medication (13%), rather than psychiatrists’ concerns about side effects (10%) (10). Additionally, the requirement for regular monitoring via venipuncture and issues related to the availability of lithium in Saudi Arabia were also noted as key obstacles (53.7% and 45.6%, respectively), highlighting the logistic challenges involved in lithium treatment. These concerns indicate that psychiatrists remain cautious due to lithium’s side effect profile and the need for frequent monitoring, which can be resource-demanding and inconvenient for patients. In addition, the perception of lithium shortage or unavailability in Saudi Arabia could encourage clinicians toward prescribing alternatives that may not be as effective in all cases. Despite the reasons for not prescribing lithium identified in our study, it is crucial to consider what is known about lithium in the literature. For instance, lithium is highly effective in clearly defined cases of bipolar I and well-characterized bipolar II; however, it tends to be less beneficial when the diagnosis is vague or overlaps with anxiety-related conditions or personality disorders involving emotional dysregulation (29).

Although lithium is still considered the first-line treatment for BD despite its side effects and the regular need for monitoring, one-quarter of the participants in our study perceived the availability of other effective mood stabilizers as a reason for not prescribing lithium. This finding raises concerns as only 15% of the participants consider lithium as a first option for the maintenance treatment of BD. Focusing on the side effects of lithium and the need for frequent monitoring while overlooking its benefits may contribute to this low rate. However, the need for monitoring lithium levels could be considered an advantage rather than a limitation, as monitoring lithium levels allows for careful titration, improved tolerability, and ensured adherence (29).

Moreover, the survey showed that the first-line treatment option for the maintenance of BD in men was valproate, followed by antipsychotics, whereas the order was reversed for women. However, these alternative medications carry significant risks, as antipsychotics are linked to substantial weight gain, have a worse metabolic profile than lithium (30, 31), and can lead to extrapyramidal symptoms and sexual dysfunction (32). Valproate has a high teratogenic risk, which makes it unsuitable for women of childbearing age (33). Moreover, the overall cost-effectiveness of lithium is superior to valproic acid and antipsychotics (34). The lack of experience or knowledge may contribute to reliance on newer medications or alternatives that may not offer the same level of efficacy for some patients with BD. Another factor for underutilization of lithium may be related to the influence of the pharmaceutical industry, i.e., marketing of antipsychotics as putative “mood stabilizers.” Antipsychotics are patentable, whereas lithium, a natural element, is not (35).

In our study, prescription practices varied by healthcare settings. Participants working in general hospitals were significantly more likely to prescribe lithium than those working in outpatient clinics. This may be due to the more complex and severe cases treated in general hospitals, where the efficacy of lithium as a mood stabilizer has become more apparent. Additionally, general hospitals might provide better resources for patient monitoring of factors such as lithium levels, which could reduce concerns over potential side effects. A multinational study reported that clinicians working in developing economies and the private sector were less inclined to choose lithium for BD maintenance (10). However, additional analysis revealed that these variations in prescription preferences did not lead to notable differences in the recommended frequency of lithium monitoring, indicating that monitoring practices remained consistent across these settings (10).

This study also found that participants with limited experience or unfamiliarity with lithium were less likely to prescribe lithium. This finding is consistent with that of a Canadian study in which older clinicians were more inclined to prescribe lithium (14), which could be attributed to their greater experience and knowledge. This observation highlights the importance of ongoing medical education and training on the use of lithium, management of its side effects, and monitoring protocols to ensure that clinicians are comfortable prescribing it.

Another factor that influenced lithium prescription in our study was patient preference. Clinicians were more likely to initiate lithium for patients who refused lithium treatment. This finding does not align with a worldwide study in which the most common reason for not choosing lithium was patients’ negative beliefs and attitudes toward the medication (10). However, we speculate that the patient’s refusal could be indicative of greater illness severity and thus reduced insight. Therefore, psychiatrists may be more inclined to prescribe lithium despite the patient’s initial reluctance, as impaired insight may lead to poor decision-making.

Our study also revealed that a significant proportion of the participants adhered to formal protocols for monitoring lithium treatment and its side effects. This finding aligns with the rate identified in an international survey, where most respondents reported using guidelines or institutional protocols for monitoring (36). Considering the well-established guidelines for lithium therapy, the percentage of participants who did not follow monitoring protocols raises concerns (19). Further assessment of the reasons for not following these guidelines should be explored. Internationally, healthcare professionals have identified personal experience and their own practice as the primary reasons for not adhering to the guidelines (36).

To the best of our knowledge, this is the first study in Saudi Arabia to assess psychiatrists’ attitudes toward prescribing lithium. This study provides valuable insights into the factors influencing lithium prescription practices in BD management. However, this study had several limitations that should be considered when interpreting the results. First, participants may have selected responses that were more consistent with the guidelines rather than reflecting on their actual practices. However, the anonymity of the survey likely encouraged honest and genuine responses. Second, the relatively low response rate and small sample size limit the generalizability of the results. Furthermore, this study involved psychiatry trainees who were still in training but had not yet practiced independently. While this could be seen as a strength, it also means that they may not have had real-world experience in which they are the sole decision-makers in choosing which medication to prescribe. Consequently, their responses might differ once they practice independently. Finally, the survey used in this study, which was adapted from an earlier study, was slightly modified by the research team, as elaborated in the methods section, to fit the local Saudi context. However, it is essential to highlight that the modified version was not piloted, and its internal consistency was not assessed. We encourage future studies to address this issue more rigorously, such as by testing a modified version of the pilot sample.

## 5 Conclusion

Although lithium remains a key treatment option for BD, its use is frequently affected by concerns about its side effects, the need for regular monitoring, and its availability in Saudi Arabia. Factors such as psychiatrists’ familiarity with lithium and their practice settings play crucial roles in influencing prescription decisions.

Future initiatives should aim to overcome barriers to prescribing lithium. One way is to enhance clinician training through standardized education on lithium use during psychiatry residency and educational workshops. Another method is to improve access to lithium to ensure its consistent availability in healthcare settings in Saudi Arabia. The third method is to develop national treatment guidelines for lithium. Such steps, among others, may promote more confident, evidence-based prescriptions practice and improve outcomes for patients with BD in the country.

## Data Availability

The raw data supporting the conclusions of this article will be made available by the authors, without undue reservation.
